# Anti-protozoal activity of extracts from chicory (*Cichorium intybus*) against *Cryptosporidium parvum* in cell culture

**DOI:** 10.1038/s41598-019-56619-0

**Published:** 2019-12-31

**Authors:** Ian David Woolsey, Angela H. Valente, Andrew R. Williams, Stig M. Thamsborg, Henrik T. Simonsen, Heidi L. Enemark

**Affiliations:** 1 Norwegian Veterinary Institute, Department of Animal Health and Food Safety, Oslo, Norway; 2 Department of Veterinary and Animal Sciences, Faculty of Health and Medical Sciences, University of Copenhagen, Frederiksberg, Denmark; 3 Department of Biotechnology and Biomedicine, Technical University of Denmark, Lyngby, Denmark

**Keywords:** Biologics, Parasitology

## Abstract

*Cryptosporidium* spp. are responsible for severe public health problems and livestock production losses. Treatment options are limited to only one drug available for human and bovine cryptosporidiosis, respectively, and both drugs exhibit only partial efficacy. Sesquiterpene lactones (SL) are plant bioactive compounds that function as a defence mechanism against herbivores. SL have demonstrated anti-parasitic properties against a range of parasitic taxa but knowledge about their anti-*Cryptosporidium* efficacy is limited. The effect of SL-rich leaf and root extracts from chicory (*Cichorium intybus* cv. Spadona) was investigated using human colon adenocarcinoma (HCT-8) cells infected with *Cryptosporidium parvum*. *C*. *parvum* oocysts were inoculated onto the cell monolayer and i) incubated for 4 hours with extracts (leaf and root extracts 300, 150, 75, 37.5, 18.75 and 9.375 μg/mL) in triplicates followed by incubation in bioactive free media (sporozoite invasion assays) or ii) incubated for 4 hours in bioactive free media followed by 48-hours incubation with extracts (growth inhibition assays). Extract toxicity on HCT-8 cells was assessed via water-soluble tetrazolium (WST)-1 assay prior to quantifying parasitic growth via immunofluorescence. Both extracts demonstrated dose-dependent inhibition in the growth inhibition assays (*p* = < 0.0001 for both extracts) but not in the invasion assays. Anti-parasitic activity did not appear to be solely related to SL content, with the extract with lower SL content (leaf) exhibiting higher inhibition at 300 μg/ml. However, given the limited treatment options available for *Cryptosporidium* spp., our study encourages further investigation into the use of chicory extracts to identify novel active compound(s) inhibiting these protozoa.

## Introduction

Worldwide, protozoan parasites belonging to the genus *Cryptosporidium* cause severe public health problems and significant production losses in the livestock industry due to persistent diarrhoea and enteritis^1^. *Cryptosporidium* is the most common cause of waterborne parasitic protozoan outbreaks^2^, and cryptosporidiosis in young children is considered the second greatest cause of mortality and diarrhoea after rotavirus^3,4^. Similarly, bovine cryptosporidiosis is recognised as an endemic cause of calf enteritis throughout the world^5^. Other symptoms include abdominal pain, nausea, vomitus, anorexia, weight loss and dehydration^6^. The symptoms are normally self-limiting with a duration of few weeks in immunocompetent hosts. However, chronic diarrhoea and spreading to extra-intestinal locations can be seen in immune-compromised hosts^7^, and the infection is associated with developmental problems, failure to thrive and malnutrition in children, mainly in non-industrialised countries^8^. The environmentally resistant *Cryptosporidium* oocysts are transmitted via the faecal-oral route directly through contact with faeces from infected hosts, or indirectly via environmental contamination or consumption of contaminated water or food. More than 30 *Cryptosporidium* species have been described so far^9^. Of these, around 12 species have been registered in humans.

*Cryptosporidium* oocysts are resistant to most commonly used disinfectants^10,11^, no vaccines are available for prevention of cryptosporidiosis in humans or livestock, and despite the severe problems caused by this pathogen, the treatment options are insufficient. Currently, nitazoxanide (Alinia^®^) is the only drug approved in the United States for use in humans^12^, but this drug exhibits only partial efficacy in reducing disease severity and oocyst shedding in immunocompetent individuals, and has little effect in immunocompromised patients^13,14^. Likewise, only one drug, halofuginone lactate (Halocur^®^), has been approved for prevention and treatment of cryptosporidiosis in calves. However, this drug has an extremely narrow therapeutic index and does not completely prevent or cure the disease^1,5^. So far, no drugs have been licensed for treatment of cryptosporidiosis in other livestock animals or pets. This situation has led to increased interest in the use of bioactive compounds to explore alternative options for treatment of cryptosporidiosis^15,16^.

The antiparasitic properties of plant bioactive compounds have been subject to intense research interest in recent years, particularly in gastrointestinal helminths as part of the ongoing effort to mitigate the spread of anthelmintic resistance in ruminants^17^. One such group of bioactive compounds, the sesquiterpene lactones (SL), are a diverse class of terpenoids with the largest diversity found in the Asteraceae family. In conjunction with antimicrobial, antiviral, antibacterial and antifungal activities^18^, SLs isolated from chicory have demonstrated antiparasitic properties^19^.

The perennial herbaceous plant chicory (*Cichorium intybus*), widely grown as forage for livestock and for human consumption, contains SL in its leaves and roots^20,21^. Extracts from these sources have demonstrated inhibition of numerous nematode species of pigs and ruminants both *in vitro* and *in vivo*^21,22^. While several investigations into the effect of SL-enriched extracts from chicory on nematodes have been conducted, potential effects of chicory on *Cryptosporidium* (and protozoa more generally) are lacking although a few studies *in vitro* have demonstrated that SL do indeed possess anti-protozoan activity against *Trypanosoma* spp, and *Leishmania* spp.^23–25^.

Using cell culture, the lifecycle of *Cryptosporidium* can be replicated in the laboratory and the different parasite stages can then be exposed to various compounds *in vitro* so that the comparative growth may be determined^26–28^. Using this method, we here assess the anti-*Cryptosporidium* potential of two distinct SL-enriched extracts obtained from the roots and leaves of cultivated *C*. *intybus* cv. Spadona against the zoonotic *C*. *parvum*.

## Materials and Methods

### Parasite material

*Cryptosporidium parvum* oocysts (Iowa strain) were purchased from Bunch Grass Farm (ID, US) and stored in 50 mL phosphate buffered saline (PBS) with penicillin (1000 IU) and streptomycin (1000 μg) at 2 × 10^7^/mL (stock solution). According to the manufacturer, the oocysts were shed 14 days prior to delivery to the institute. The oocyst viability was assessed to 82% via 4’,6-diamidino-2phenylindole/propidium iodide (DAPI/PI) stained oocysts in wet mounts immediately prior to inoculation of the first assay plate^29,30^ (see supplementary material). All assays were conducted within 10 days. Oocysts for inoculation of the plates were taken directly from the stock solution and prior to performing the assays the oocyst concentration was determined by diluting 10 μL stock solution with 990 μL PBS and counting three repeats after staining with Crypt-a-Glo (Waterborne Inc, LA, USA).

### HCT-8 cell culture

Human colon adenocarcinoma (HCT-8) cells (ECACC, Salisbury, UK) were cultured in maintenance medium (RPMI-1640, Biowest, France) supplemented with horse serum (5% *v/v*), foetal bovine serum (FBS) (5% *v/v*), 1 mM sodium pyruvate and an antibiotic/antimycotic solution (penicillin (100 U/mL), streptomycin (100 μg/mL) and amphotericin B (0.25 μg/mL) (all Sigma-Aldrich, MO, USA). Cell sub cultivation was performed twice per week with trypsin/EDTA (Sigma Aldrich, MO, USA). Cells were incubated at 37 °C, in 5% CO_2_.

### Plant materials

Chicory (*Cichorium intybus* cv. Spadona) (DSV Ltd., Denmark) was sown as pure sward (7 kg seeds/ ha) in May 2017 and harvested September 2017, at field facilities of University of Copenhagen (Taastrup, Denmark, 55_6704800N, 12_2907500E). Several plants were harvested, the leaves were frozen to −80 °C, freeze-dried for 48 hours and then dry blended. Roots were dug up, grinded and then freeze dried at −80 °C. All material was stored at −20 °C until extraction.

### Sesquiterpene lactones enriched extraction method

An extraction procedure optimized for recovery of SL was performed as described by Ferioli and D’Antuono^31^ and Peña-Espinoza *et al*.^21^. Dry plant material was dissolved in 15 mL of 2% (v/v) formic acid in MeOH/H20 (4/1; v/v), vortexed for 1 min, sonicated for 10 min at room temperature (RT) and centrifuged at 2647 × g for 10 min (repeated three times). Extracts were dried under reduced pressure at 35 °C and freeze dried for 30 minutes. Dried extract was recovered with Viscozyme^®^ L cellulolytic enzyme mixture in Citrate-Phosphate buffer pH 5.4, Sigma-Aldrich (Concentration 10 mg/1 mL buffer). For complete removal of bound sugars, the mixture was left overnight at 37 °C. For collection of metabolites and removal of free sugar, ethyl acetate was added to the aquatic phase followed by centrifugation for phase separation. The collected ethyl acetate phase was removed under reduced pressure (35 °C) and the extract was dissolved in 14% methanol in dichloromethane and further purified by solid phase extraction (SPE). A SPE vacuum manifold was equipped with 12 × 6 mL SPE tubes (Supelclean® LC-Si SPE tubes, Supelco 505374). SPE cartridges were conditioned with 6 mL dichloromethane/i-propanol (1/1;v/v) and equilibrated with 6 mL dichloromethane. The extract was loaded in each SPE tube and the obtained liquid fractions were transferred into one glass flask. Collected fractions were dried under nitrogen flux and the resulting purified extracts were weighed.

Extracts used for *in vitro* assays were suspended in 100% dimethyl sulfoxide (DMSO) at a concentration of 100 mg dry weight extract/mL DMSO and stored at −20 °C until use. For chemical analysis extracts were suspended in MeOH at a concentration of 10 mg dry weight extract/mL methanol.

### Quantification and identification of sesquiterpene lactones

#### HPLC-MS QQQ

Quantification of SL was performed using an Agilent Infinity 1290 UHPLC system QQQ (Agilent Technologies, Santa Clara, CA, USA) with a diode array detector. In a Phenomenex Kinetex C18 (150*2.1 mm, 2.6 μm) column. The gradient was linear with water and acetonitrile (both buffered with 20 mM formic acid) starting at 10% at a flow rate of 0.59 mL/min and ending at 10 min (100%). The ion source was Agilent Jet Stream Electrospray Ionization with Dynamic MRM as scan type and an injection volume of 1 μL was used. MS detection was performed on an Agilent 6490 Triple Quad MS equipped with Agilent Dual Jet Stream electrospray ion source with Dynamic MRM. Identification of individual compounds was based on findings in Graziani *et al*.^20^. Standards used for quantification was lactucin (3809, Extrasynthese SAS, 69727 GENAY Cedex) and 11beta,13-dihydrolactucopicrin (3811, Extrasynthese SAS, 69727 GENAY Cedex). A dilution curve of 0.1, 1, 10 and 100 ug/mL was used to calculate the equation.

### Parasite inoculation onto cell monolayer

HCT-8 cells were seeded onto Nunclon^®^ 96-well plates (Sigma Aldrich, MO, USA) (2 × 10^5^ cells/well) and incubated in maintenance medium for 24 hours until they reached confluence. Prior to infection of the cell monolayer, the oocysts were pre-treated as described by Slifko *et al*.^32^. Briefly, 100 μL oocyst stock solution (2 × 10^6^ oocysts) was suspended in 10.5% bleach solution (800 μl MilliQ and 100 μl 5.25% sodium hypochlorite) for 10 min. The suspension was subsequently centrifuged at 4000 × g for 4 min at 4 °C and the supernatant aspirated. The oocysts were then washed in 10 mL MilliQ water and centrifuged again (4000 × g, 4 min, 4 °C) and re-suspended in 19.8 mL maintenance medium pre-warmed to 37 °C. The oocyst suspension was vortexed and 100 μL (≈10,000 oocysts) were added to the cell monolayer in each well and incubated for 4 hours (37 °C, 5% CO_2_). For all assays, the border wells of each 96-well plate were excluded as host cells and parasites in these wells may experience increased stress due to atmospheric oxygen diffusing into the medium and inhibiting the outer wells more than those located centrally^28^. HCT-8 cells were on their 8^th^ passage prior to use in the assays.

### Extract preparation

The chicory extracts (100 mg/mL) were diluted to 30 mg/mL and serially diluted in DMSO yielding 6 concentrations (30, 15, 7.5, 3.75, 1.875 and 0.9375 mg/mL). Maintenance medium was added at 1:100 dilution yielding 300, 150, 75, 37.5, 18.75 and 9.375 μg/mL in 1% DMSO.

### Parasite growth inhibition assay

**Figure 1:**
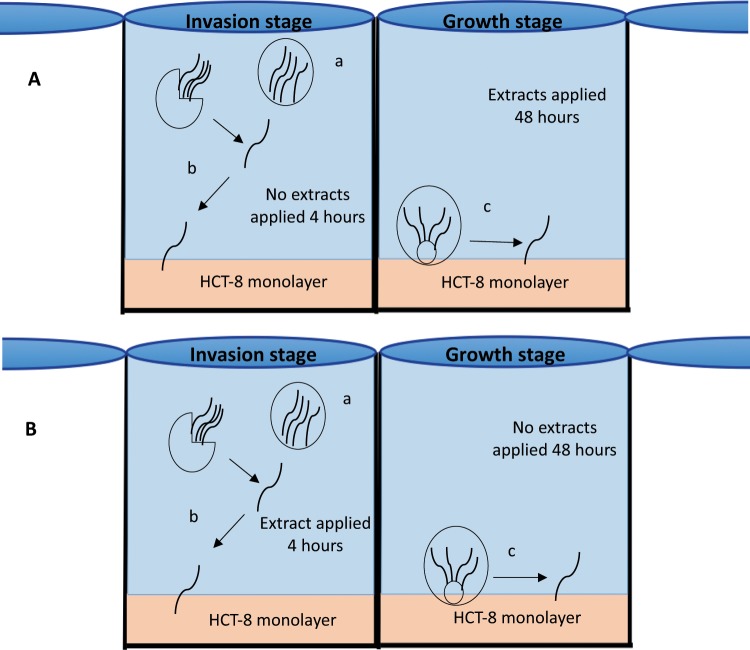
Schematic illustration of parasite exposure to extracts during the growth inhibition assays (**A**), parasite exposure to extracts during the sporozoite invasion inhibition assays (**B**) (a, oocysts containing 4 sporozoites, b, oocyst excysting and sporozoites invading the cell monolayer and c, parasite growth in the cell monolayer) (adapted and used with permission from^16^).

After incubation of the parasites on the HCT-8 cell monolayer for 4 hours, maintenance medium was aspirated from each well and the cell monolayers washed three times in 100 μL PBS. Extracts were then added to each well (100 μL/well) (Fig. 1a). In wells containing inactivated oocysts (placed in water bath at 70 °C for 30 min), used as controls for the washing steps of the assay, blank wells (no oocysts), used to assess non-specific background fluorescence, and negative controls (viable oocysts with no extract), maintenance medium with 1% DMSO was added. As a positive control the antibiotic paromomycin (500 μg/mL), previously demonstrated to inhibit *C*. *parvum in vitro*^33^ was added to the maintenance medium. Oocysts were then added to the monolayer and incubated for 48 hours (37 °C, 5% CO_2_). All extracts and controls were tested in triplicates, and the assay was repeated twice.

### Sporozoite invasion inhibition assay

Maintenance medium was aspirated from the 96-well plate and washed twice in PBS (100 μL). Extracts and control (same as per the parasite growth inhibition assay) substances were added to the well monolayers and subsequently oocysts were added and incubated for 4 hours (37 °C, 5% CO_2_). Extract and control solutions were then aspirated and washed three times with 100 μL PBS after which 100 μL maintenance medium was added to each well and incubated for 48 hours (37 °C, 5% CO_2_) (Fig. 1b). Triplicate samples of all extracts and controls were conducted and the assay was performed twice.

### Cell viability

Fresh maintenance medium solution containing 10% (*v*/*v*) water-soluble tetrazolium salt (WST-1, Roche Diagnostics GmbH, Vienna, Austria) (100 μL) was added to each well after 48-hour incubation. The plates were subsequently incubated at 37 °C and the optical density measured at 450 nm (OD_450_). Plates were read every 15 minutes until the negative control wells reached an OD_450_ value > 1. Cytotoxicity was defined as cell proliferation <75% of the negative control wells^28^.

### Parasite immunodetection

Cell monolayers were washed with 100 μL PBS (×3) and fixed in 100 μL ice-cold methanol. As per manufacturer’s instructions, Sporo-glo (Waterborne Inc, LA, USA), a fluorescein-labelled rat anti-*C*. *parvum* sporozoite polyclonal antibody, was added to each well and incubated in a humid chamber at room temperature in the dark for 45 minutes. Sporo-glo was aspirated from each well washed with 100 μL PBS (×3) and counterstain added. After 1 min the counterstain was removed, cells were washed once more and one drop of mounting medium added to each well. Monolayers were then viewed under a fluorescence microscope (AX10, Zeiss, Germany) at ×200 magnification, 480-nm excitation and 550-nm emission. Examples of negative control well raw images and post processing analysis are available in the supplementary material.

### Parasite quantification

For each well 40 photographs (corresponding to 45.8% of the well surface area) (AxioCam 503, Zeiss, Germany) were obtained systematically (20 on the well periphery and 20 centrally) to ensure no parasite stages were counted more than once. Images were subsequently processed to obtain the number of fluorescent particles corresponding to the sizes of the various intracellular life cycle stages of the parasite^26^ using the batch process function in ImageJ (NIH, Bathesda, MD, USA). This allows the program to analyse multiple image files in rapid succession. A macro (coded instructions) adapted from Bessoff *et al*.^34^ was used to instruct the program to only count fluorescent particles corresponding to the various life cycle stages of *Cryptosporidium* (see supplementary material). Counts from stained monolayers with no parasite (blanks) were subtracted from wells inoculated with oocysts to control for background noise. *C*. *parvum* growth was assessed for each treatment as a percentage in relation to the mean of negative control wells, defined as 100% parasite development.

### Statistical analysis

Half maximal inhibitory concentrations (IC_50_) were calculated for each extract for both the growth inhibition and the sporozoite invasion assays using non-linear regression and IC_50_ of two different extracts compared by extra-sum-of-squares F-test (GraphPad Prism version 8.00). The average well counts for each extract/concentration were assessed as a percentage of relative growth against the negative control wells. One-way ANOVA and linear regression analysis were performed on counts per photograph per well against concentration for each extract. Data were analysed in R Studio 1.0.143. Values were considered significant if *p* = < 0.05.

## Results

**Figure 2:**
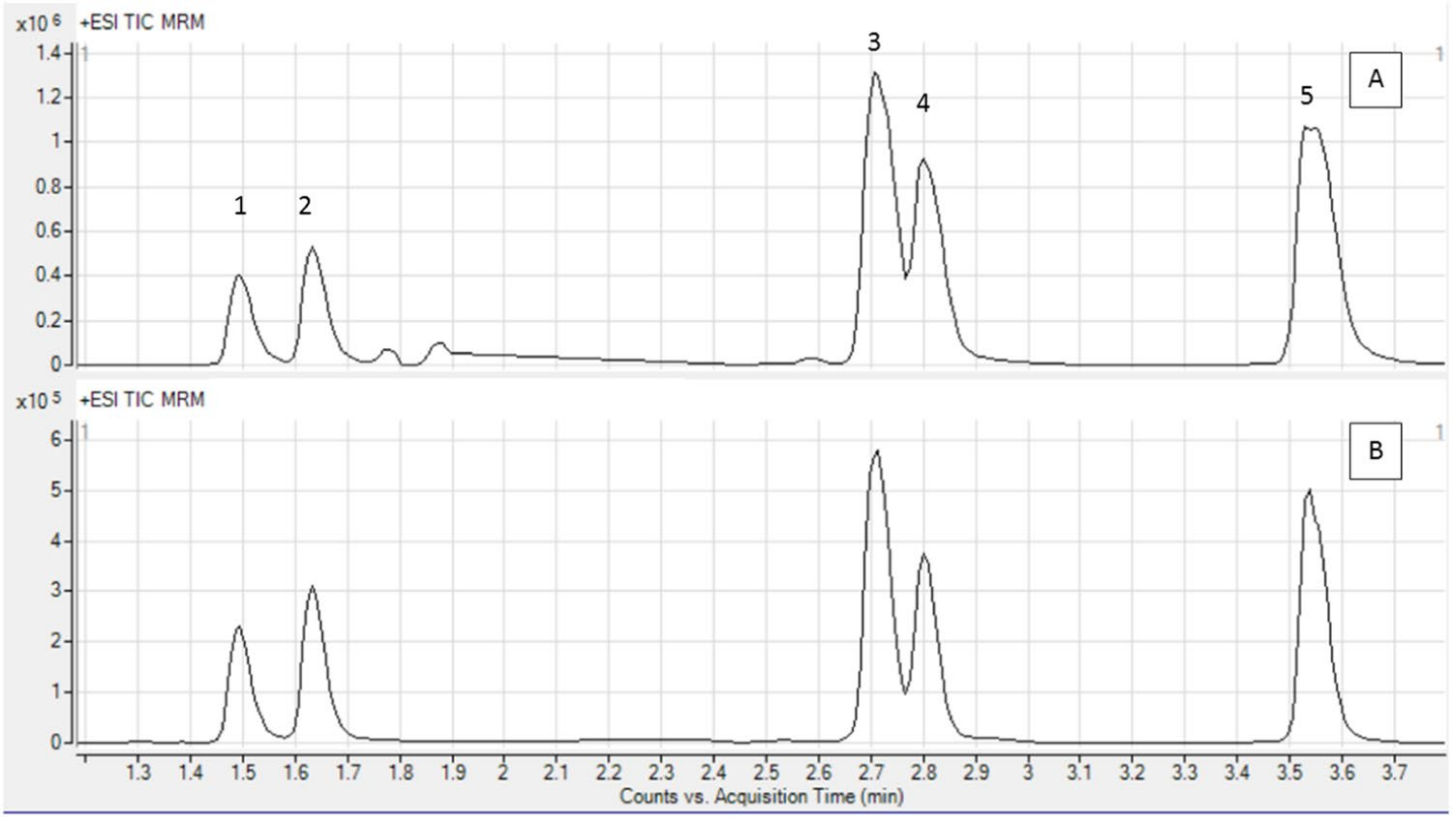
Peaks detected by high performance liquid chromatography coupled with triple quadrupole mass spectrometry (HPLC-MS QQQ) identification of six sesquiterpene lactones from chicory (*Cichorium intybus*). Lactucopicrin and 11beta,13-Dihydrolactucopicrin are very similar in size and charge, hence, their peaks overlapped. The total content of the two is therefore calculated from peak 5. The individual compounds 1–5 are further described in Table 1. (**A**) leaf extract. (**B**) root extract. Please note the different scales on the Y-axes.

Quantification of the individual compounds detected by HPLC-MS QQQ demonstrated that the amount of SL in the leaves was 20.51 mg/g in the dry extract, while the content in the roots was 52.284 mg/g, representing a 2.55 times higher amount of SL in the roots than the leaves. The distribution of the six main SL was broadly similar for the extracts of the two tissue types (Table 1). All compounds detected belong to the guainolides, a subgroup of SLs that are the most abundant and diversified^35^. These six compounds are the main SL present in chicory extracts and potentially the active compounds associated with anthelmintic activity^21^. The compounds were quantified to determine potential association with anti-parasitic activity. It was not possible to separate lactucopicrin and 11-β,13-dihydrolactucopicrin since the size and charge of these two compounds are very similar, hence their peaks overlapped. The total content of the two is therefore calculated from peak 5 (Fig. 2).

**Table 1:** Amount of individual sesquiterpene lactones (SL) in chicory leaf and root dry extracts (mg/kg). Quantification was based on standards from extrasyntase, lactucin and 11beta,13-Dihydrolactucopicrin. The standard curve was based on the concentrations 0.1, 1, 10 and 100 µg/mL.

Compound (peak number)	Spadona leaf		Spadona root	
	Amount of compound [mg/kg in dry extract]	Distribution [%]	Amount of compound [mg/kg in dry extract]	Distribution [%]
Dihydrolactuicin (1)	2681.46	10.52	4985.99	7.36
Lactucin (2)	3708.30	14.54	6620.31	9.78
8-Deoxy-lactucin (3)	7706.57	30.22	20141.68	29.75
11beta,13-Dihydro-8-deoxy-lactucin (4)	4511.11	17.69	14658.27	21.65
11beta,13-Dihydrolactucopicrin/Lactucopicrin (5)	1903.08	27.03	5878.38	31.45
Total	20510.52	100	52284.62	100

### Parasite growth inhibition assay

**Figure 3:**
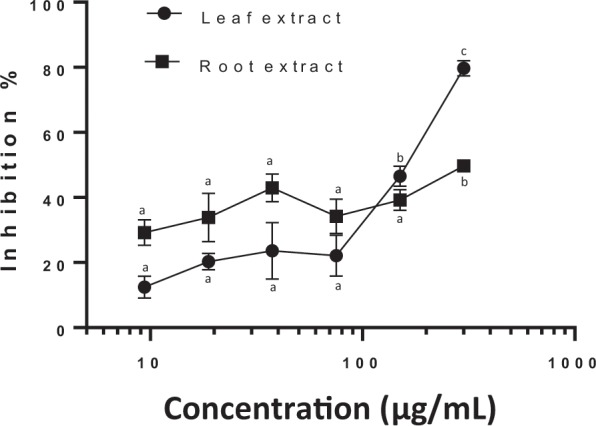
Dose-dependent growth inhibition of *Cryptosporidium parvum* by chicory leaf and root extracts. Data presented are the mean of two independent parasite growth inhibition assays, with three replicate wells per assay (mean ± inter-experiment S.D.). Letters indicate significant differences in inhibition between concentrations of the same extract. All concentrations for both extracts exhibited significantly reduced parasite counts compared to negative control wells with the exception of 9.375 µg/ml in the leaf extract.

**Figure 4:**
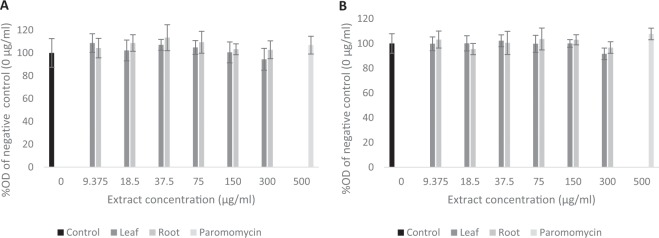
Cell viability after exposure to extracts at varying concentrations expressed as % OD value of negative controls (100% viability). (**A**) Growth inhibition assay (**B**) Sporozoite invasion assay (mean ± inter-experiment S.D).

Both extracts exhibited concentration dependent inhibition of *C*. *parvum* growth (One-way ANOVA, *p* = < 0.0001 for both extracts) up to 79.68 ± 5.36% compared to the negative control growth for leaf and 49.74 ± 3.41% for root at 300 μg/mL (Fig. 3). The lowest concentration with significantly reduced growth compared to the negative control was 18.75 μg/mL for the leaf extract (*p* = 0.0180) and 9.375 μg/mL for the root (*p* = 0.0006). Leaf extract was more inhibitory than root at 300 μg/mL (*p* = < 0.0001) but significantly less inhibitory at 9.375 μg/mL (*p* = 0.0089). For the root extract, 300 μg/mL was significantly more inhibitory than 150 μg/mL (*p* = 0.0143), with this concentration not significantly more inhibitory than lower concentrations. However, for the leaf extract both 300 μg/mL (*p* = < 0.0001) and 150 μg/mL (*p* = 0.0059) demonstrated significantly reduced growth compared to lower concentrations. The IC_50_ values were significantly different, 139.7 μg/mL for leaf and 660.3 μg/mL for root extracts (comparison by F-test: *p* = < 0.0001). Paromomycin at 500 μg/mL wells exhibited inhibition of 88.89 ± 1.55 (*p* = < 0.0001) compared to the negative control wells and was significantly more inhibitory than all concentrations of the root extract, however, this was not the case for the leaf extract, with the highest two concentrations being statistically as inhibitory as paromomycin. No cytotoxic effects of either extract were observed in any of the wells for both assay plates (Fig. 4).

### Sporozoite invasion inhibition assay

**Figure 5:**
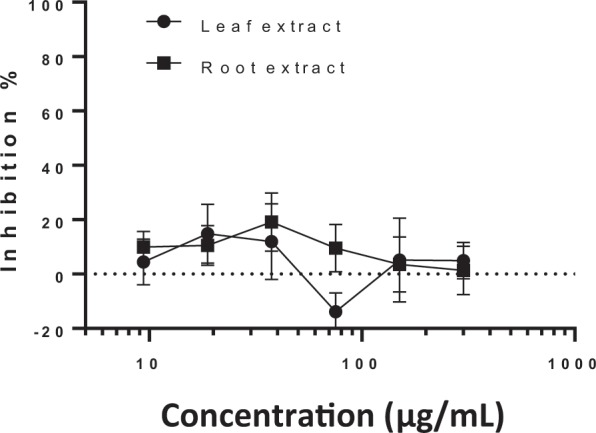
*Cryptosporidium parvum* invasion inhibition by chicory leaf and root extracts. Data presented are the mean of two independent invasion assays, each performed with three replicate wells per assay (mean ± inter-experiment S.D.).

Concentration dependent inhibition was not observed for either leaf and root extracts (One-way ANOVA *p* = 0.625 for leaf and *p* = 0.776 for root extract). With no extracts exhibiting significantly reduced counts in comparison to negative control wells and no curve could be fitted (Fig. 5). Paromomycin exhibited no significant inhibition in comparison to the negative control wells (*p* = 0.976), nor any other concentrations. None of the extracts appeared to exert any toxic effects on the cells (Fig. 4).

## Discussion

This study demonstrated concentration-dependent inhibition of *C*. *parvum* for both chicory (cv. Spadona) leaf and root extracts in the growth inhibition assays but not in the sporozoite invasion assays.

Paromomycin at 500 μg/mL exhibited 88.15 ± 2.26% *C*. *parvum* growth inhibition. In accordance with our findings, values in the literature are quite variable. A previous study exhibited an IC_50_ concentration of 711 μM (437.75 μg/mL)^36^. Perkins *et al*.^33^. demonstrated *in vitro* that the IC_50_ of paromomycin is 85 μM (52.3 μg/mL) against *C*. *parvum* with 400 μM (246.3 μg/mL) inhibiting *in vitro* growth by 52% although variation was high with inhibition reaching approximately 90%. In contrast, 200 μg/mL paromomycin in a separate study reduced *C*. *parvum* growth by 38% with 1 mg/ml inhibiting growth by 60%^37^. Inhibition by 500 μg/mL paromomycin was not observed in the sporozoite invasion assays (*p* = 0.0976). This differs from the observed effect of nitazoxanide, where a 2-hour incubation of the drug in a similar experimental design inhibited parasite development >70% at 50 μg/mL^37^.

Previous studies investigating differences between plant extracts on the invasion and growth of *C*. *parvum* have yielded different outcomes. Curcumin application resulted in inhibition of sporozoite invasion and growth with greater inhibition occurring during growth inhibition. In that study, 73.7 μg/mL curcumin inhibited parasite numbers by 65% during sporozoite invasion as opposed to a > 95% inhibition at 18.4 μg/mL during growth inhibition^27^. Conversely, no effect on sporozoite invasion was observed in similar assays assessing the effect of oregano essential oil despite significant inhibition during the intracellular growth phase^16^. Although no inhibition of the parasite was found during sporozoite invasion, high variability exhibited by cell culture assays determining *C*. *parvum* infectivity and inactivation have demonstrated that such assays are only suitable for discerning relatively large effects on these parasites^38^. The effect of limited exposure of the parasite to the extract, coupled with inherent variation in the oocyst inoculate could be too slight for the fidelity of this assay design. Oocysts tend to clump together which in turn will increase sedimentation rate. Thus, achieving a uniform inoculate of oocysts between wells is challenging^38,39^. This likely explains the fluctuation above and below 0% inhibition in the sporozoite invasion assays (Fig. 5), but supports data generated from the growth inhibition assays, with uniform trends in inhibition occurring in both repeated plates.

The 2.5-fold difference in SL content between leaf and root extracts and the increased activity of the leaf extract at 300 μg/mL when compared to root at 300 μg/mL (Fig. 3) suggests that SL are either not the only anti-protozoal compounds present in the chicory extracts or exert no inhibition on the parasite. It would be expected that significantly more inhibition would occur in response to the root extract if the parasite was responding solely to the SL content, especially considering that the specific SL are present in roughly similar proportions in both extracts. It should be noted that in a recent study, significantly higher quantities of guaianolide oxalates were found to be present in leaves than roots^35^ however the SL extraction process used in the current study removed all oxalate forms of the guaianolides and as such, cannot be responsible for anti-*Cryptosporidium* activity observed. Although concentration dependent inhibition occurs for the root extract in the range used for this study, the inhibition curve is flat compared to the leaf extract with 95% CI for IC_50_ 196.7-51,8847 rendering the IC_50_ value unreliable. Significantly greater inhibition at 300 μg/mL compared to 150 μg/mL with all other concentrations being similarly inhibitory suggests that increasing the concentration range may enable accurate IC_50_ calculation, but this needs to be considered with a view to cytotoxic effects on the host cells. The leaf extract at 300 μg/mL was more inhibitory than at 150 μg/mL with this concentration in turn demonstrating greater inhibition than all other concentrations. However, at 9.375 μg/mL this root was more inhibitory than leaf which could be indicative of each extract containing different inhibitory compounds.

Inhibitory effects of chicory on parasitic nematodes in ruminants and mono-gastric livestock is well documented and convincing evidence from *in vitro* studies suggest that SL are the primary compounds responsible for this inhibition^40^. Anthelmintic activity of chicory was demonstrated in an *in vitro* egg hatch inhibition assay against *Haemonchus contortus*. Lactucin appeared to play a minimal role in inhibition and it was likely that 8-Deoxy-lactucin was responsible for the observed activity^41^. Similarly, 8-Deoxy-lactucin:lactucin compound profiles have also shown inhibition *in vitro* against *Ostertagia ostertagi* adult stages^21^. SL profiles of both extracts used in this study have a high 8-Deoxy-lactucin:lactucin ratio, which suggests that if there are specific SL compounds acting upon *C*. *parvum*, they are distinct from those which exhibit anthelmintic effect. These two SL compounds have a similar distribution in the root and leaf extracts in this study, therefore, if they were responsible for inhibition we would see greater inhibition in the root extract.

SL have previously demonstrated activity against several protozoa including haemoflagellates such as *Trypanosoma* spp, *Leishmania* spp.^25,42^, and purified lactucopicrin and lactucin SL resulted in 100% inhibition of another haemoprotozoan parasite *P*. *falciparum* at 50 and 10 μg/mL respectively after 48-hour exposure. At 300 μg/mL, lactucin concentrations contained within extracts used for our study were 1.11 μg/mL for leaf and 1.98 μg/mL for root extract. At these concentrations, purified lactucin extract inhibition was >30%^19^. The concentration of lactucopricin in the extracts could not be quantified due to overlapping peaks (Fig. 2.). A recent study using extracts from leaf and root of chicory (cv. Spadona) demonstrated a clear dose dependent inhibition of *Trypanosoma cruzi* with highest levels of inhibition by the root extract (Valente *et al*. unpublished data). With different SL compounds acting upon helminths and protozoa, further investigation into the effects of individual SL against *C*. *parvum* would be welcome, especially as these studies have focused on haemoprotozoan parasites, which may respond very differently to potential inhibitory compounds than *C*. *parvum*. Isolation of the individual SL compounds should be performed to determine their anti-parasitic effects in addition to specific combinations in case of synergistic effects.

Efficacy of SL against nematodes has been reserved to abomasal parasites *in vivo*, with intestinal parasites remaining unaffected inside the host post SL treatment^43,44^. *In vitro* activity has recently been demonstrated against adult stages of *Cooperia oncophora* suggesting active metabolites are present in different concentrations within various organs of the host^45^. The prospect of other bioactive compounds being responsible for the inhibition observed in our study is therefore intriguing. As an intestinal parasite, SL activity against *C*. *parvum* could possibly be of little practical use in cattle if metabolites are not present in sufficient quantities in the target organ. It is therefore necessary to expand the investigation of active compounds in these extracts since chicory could contain yet undiscovered anti-protozoal bioactive compounds. Aside from SL, numerous other bioactive compounds have been reported from chicory such as hydroxycinnamic acids with e.g. insecticidal and acaricidal effects^40,46,47^. Such investigations would optimally be done by bioassay guided fractionation and identification of active compounds. It could be the combination of compounds that result in the greatest inhibition. Hence, a small change in the combination could result in an extract substantially more effective.

Chicory is used to improve the availability of forage and nutritive value in ruminants globally^41,48^ and is widely cultivated for human consumption, particularly its leaves for use in salads^20^. Moreover, the plant is used for various medicinal applications due to its health promoting properties such as e.g. anti-bacterial, anti-inflammatory and anti-hepatotoxic effects^49^. Based on the results of our study we may now add anti-cryptosporidial effect to this list. Regardless of the mechanisms responsible for the inhibition of *C*. *parvum*, it is an important finding that both chicory leaf and root extracts resulted in reduced growth of the parasite *in vitro*.

## Conclusion

A significant dose-dependent inhibition of both chicory leaf and root extracts in two different (replicated) assays is strongly suggestive of inhibitory effects on *C*. *parvum* and a clear uniform trend in parasite inhibition was observed in both growth inhibition assays for both extracts. Anti-parasitic activity did not appear to be solely related to SL content, with higher inhibition in the leaf extract at 300 μg/ml despite lower SL content, indicating that different compounds may be responsible for inhibition in each extract. Although it is not possible to determine the extent to which this inhibition is achieved by SL, a clear effect of chicory extracts on *C*. *parvum* is a useful/noticeable finding considering the interest of this crop in the treatment or prevention of numerous parasitic diseases. Further studies should focus on confirming this inhibition and the determination of extract efficacy on varying life cycle stages of the parasite and identification of responsible bioactive compounds. This could involve increasing assay time from 48 to 72 hours and the exposure of *C*. *parvum* to extracts at different points during growth inhibition. Successful confirmation of SL or other bioactive inhibition of these extracts *in vitro* would then need to be verified *in vivo*.

## Supplementary information

Supplementary figure 1

Supplementary figure 2

Supplementary figure 3

Supplementary figure 4
