# Supplementary material for: Anti-protozoal activity of extracts from chicory (Cichorium intybus) against Cryptosporidium parvum in cell culture
Source: Sci Rep. 2019 Dec 31;9:20414. doi: 10.1038/s41598-019-56619-0 (PMC6938481; doi:10.1038/s41598-019-56619-0)
Supplement: Supplementary file 1 — Supplementary figure 1 [file 41598_2019_56619_MOESM1_ESM.pdf]

Supplementary figure S1:

Anti-protozoal activity of extracts from chicory (*Cichorium intybus*) against *Cryptosporidium parvum* in cell culture.

Authors:
Ian David Woolsey[1*], Angela H. Valente[2], Andrew R. Williams[2], Stig M. Thamsborg[2], Henrik T. Simonsen[3] and Heidi L. Enemark[1].

1. Norwegian Veterinary Institute, Department of Animal Health and Food Safety, Oslo, Norway.
2. Department of Veterinary and Animal Sciences, Faculty of Health and Medical Sciences, University of Copenhagen, Frederiksberg, Denmark.
3. Department of Biotechnology and Biomedicine, Technical University of Denmark,

Lyngby, Denmark.

*Corresponding author:

ian.woolsey@vetinst.no

+47 92265696

Legend:
Examples of *Cryptosporidium parvum* oocysts stained with A) fluorescein isothiocyanate (FITC), B) 4',6-diamidino-2phenylindole (DAPI), or C) propidium iodide (PI) for quantification and viability assessment prior to inoculation onto cell cultures. DAPI-/PI- oocysts as shown in B and C (arrow) were categorized as potentially viable while inserts B and C show DAPI+ and PI+ oocysts, respectively. DAPI+/PI- oocysts were categorized as viable and DAPI+/PI+ as non-viable. The scale bars are equivalent to 50 µm. Photos: Heidi H Petersen, Technical University of Denmark (used with permission).

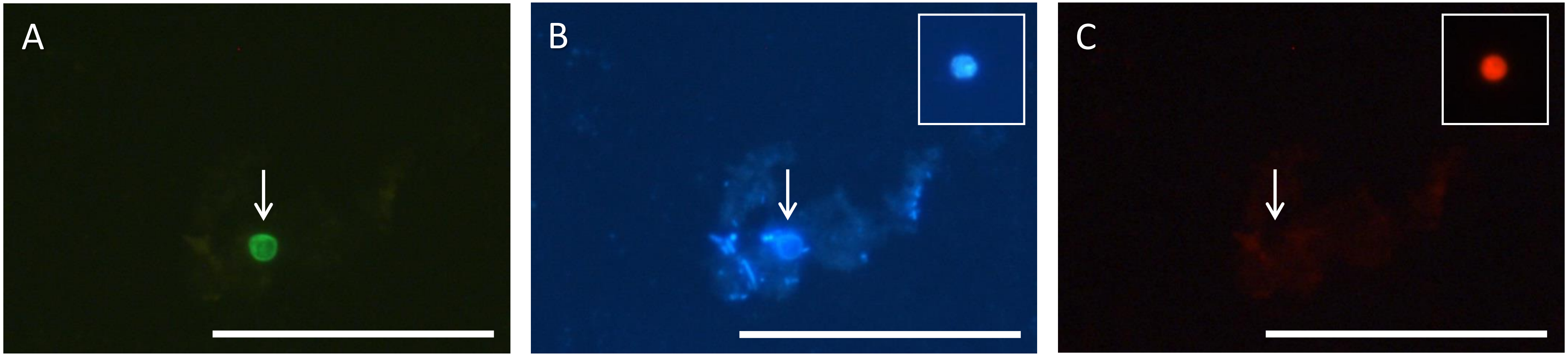
A
B
C
