# Supplementary material for: Anti-protozoal activity of extracts from chicory (Cichorium intybus) against Cryptosporidium parvum in cell culture
Source: Sci Rep. 2019 Dec 31;9:20414. doi: 10.1038/s41598-019-56619-0 (PMC6938481; doi:10.1038/s41598-019-56619-0)

**Supplementary figure S2:**

**Anti-protozoal activity of extracts from chicory (*Cichorium intybus*) against *Cryptosporidium parvum* in cell culture.**

**Authors:**

Ian David Woolsey^1*^, Angela H. Valente^2^, Andrew R. Williams^2^, Stig M. Thamsborg^2^, Henrik T. Simonsen^3^ and Heidi L. Enemark^1^.

1. Norwegian Veterinary Institute, Department of Animal Health and Food Safety, Oslo, Norway.

2. Department of Veterinary and Animal Sciences, Faculty of Health and Medical Sciences, University of Copenhagen, Frederiksberg, Denmark.

3. Department of Biotechnology and Biomedicine, Technical University of Denmark, Lyngby, Denmark.

*Corresponding author:

ian.woolsey@vetinst.no

+47 92265696

**Legend:**

Image of a negative control well containing *C. parvum* parasite growth in HCT-8 cell culture. Image taken 48 hours post infection after fixation with methanol and incubation with Sporo-glo (Waterborne Inc, LA, USA). The image was viewed with a fluorescence microscope (AX10, Zeiss, Germany) at ×200 magnification, 480-nm excitation and 550-nm emission and captured with an AxioCam 503 (Zeiss, Germany). Scale bar = 25 µm


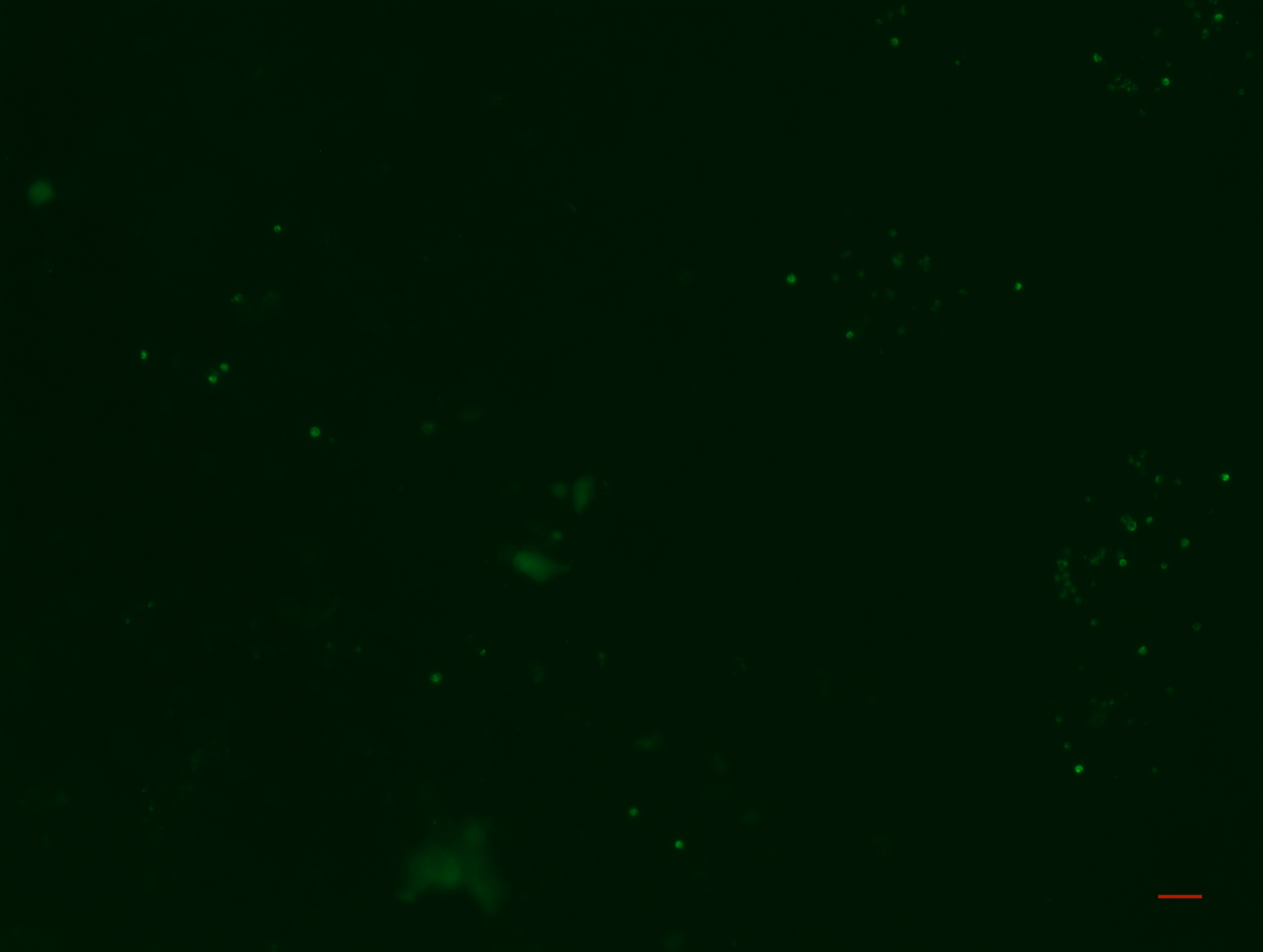

Supplement: Supplementary file 2 — Supplementary figure 2 [file 41598_2019_56619_MOESM2_ESM.docx]
