# Supplementary material for: Anti-protozoal activity of extracts from chicory (Cichorium intybus) against Cryptosporidium parvum in cell culture
Source: Sci Rep. 2019 Dec 31;9:20414. doi: 10.1038/s41598-019-56619-0 (PMC6938481; doi:10.1038/s41598-019-56619-0)
Supplement: Supplementary file 3 — Supplementary figure 3 [file 41598_2019_56619_MOESM3_ESM.docx]

**Supplementary figure S3:**

**Anti-protozoal activity of extracts from chicory (*Cichorium intybus*) against *Cryptosporidium parvum* in cell culture.**

**Authors:**

Ian David Woolsey^1*^, Angela H. Valente^2^, Andrew R. Williams^2^, Stig M. Thamsborg^2^, Henrik T. Simonsen^3^ and Heidi L. Enemark^1^.

1. Norwegian Veterinary Institute, Department of Animal Health and Food Safety, Oslo, Norway.

2. Department of Veterinary and Animal Sciences, Faculty of Health and Medical Sciences, University of Copenhagen, Frederiksberg, Denmark.

3. Department of Biotechnology and Biomedicine, Technical University of Denmark, Lyngby, Denmark.

*Corresponding author:

ian.woolsey@vetinst.no

+47 92265696

**Legend:**

ImageJ (NIH, Bathesda, MD, USA) output of suppl. 2 demonstrating particle selection by the program for parasite quantification. Scale bar = 25 µm.
