# Supplementary material for: Anti-protozoal activity of extracts from chicory (Cichorium intybus) against Cryptosporidium parvum in cell culture
Source: Sci Rep. 2019 Dec 31;9:20414. doi: 10.1038/s41598-019-56619-0 (PMC6938481; doi:10.1038/s41598-019-56619-0)
Supplement: Supplementary file 4 — Supplementary figure 4 [file 41598_2019_56619_MOESM4_ESM.docx]

**Supplementary figure S4:**

**Anti-protozoal activity of extracts from chicory (*Cichorium intybus*) against *Cryptosporidium parvum* in cell culture.**

**Authors:**

Ian David Woolsey^1*^, Angela H. Valente^2^, Andrew R. Williams^2^, Stig M. Thamsborg^2^, Henrik T. Simonsen^3^ and Heidi L. Enemark^1^.

1. Norwegian Veterinary Institute, Department of Animal Health and Food Safety, Oslo, Norway.

2. Department of Veterinary and Animal Sciences, Faculty of Health and Medical Sciences, University of Copenhagen, Frederiksberg, Denmark.

3. Department of Biotechnology and Biomedicine, Technical University of Denmark, Lyngby, Denmark.

*Corresponding author:

ian.woolsey@vetinst.no

+47 92265696

**Legend:**

The macro used in ImageJ (NIH, Bathesda, MD, USA) (adapted from Bessoff et al 2013) to enable the identification of particles corresponding to *C. parvum* life cycle stage sizes.

run("Subtract Background...", "rolling=3");

run("8-bit");

setAutoThreshold("Default dark");

//run("Threshold...");

//setThreshold(5, 255);

run("Convert to Mask");

run("Analyze Particles...", "size=48.14814814814815-146.9135802469136 pixel display include summarize");
